# Enhanced Modulation of Terahertz Generation in Optically Pumped Silicon-Based CoFeB/Ir Heterostructures

**DOI:** 10.3390/nano16090530

**Published:** 2026-04-28

**Authors:** Ruijie Peng, Zuanming Jin, Yexing Jiang, Huiping Zhang, Wei He, Yan Peng

**Affiliations:** 1 Terahertz Technology Innovation Research Institute, Terahertz Spectrum and Imaging Technology Cooperative Innovation Center, Shanghai Key Lab of Modern Optical System, University of Shanghai for Science and Technology, Shanghai 200093, China; prjshlg@163.com (R.P.); 18138200369@163.com (Y.J.); hpzhang@usst.edu.cn (H.Z.); 2 Shanghai Institute of Intelligent Science and Technology, Tongji University, Shanghai 200092, China; 3 State Key Laboratory of Magnetism, Institute of Physics, Chinese Academy of Sciences, Beijing 100190, China

**Keywords:** broadband terahertz emission, ferromagnetic heterostructure, inverse spin Hall effect, photothermal effect, spin diffusion length

## Abstract

Silicon-compatible spintronic terahertz emitters (STEs) are crucial for on-chip ultrafast optoelectronic integration, yet their all-optical controllability remains a key challenge. Here, we fabricate a Ta-buffered CoFeB/Ir heterostructure on Si substrates and realize, for the first time, the enhancement and nonlinear modulation of coherent THz emission under continuous-wave (CW) optical pumping at room temperature. The THz emission, dominated by the inverse spin Hall effect, features an ultrabroad 0–2.5 THz bandwidth and robustness against femtosecond pump fluence and polarization variations. The all-optical modulation of THz generation originates from the competition between photothermal and photodoping effects in the Si substrate. The heterostructure-side pumping with a 450 nm CW laser yields an increased modulation of 46% at 2.546 W cm^−2^ due to the photothermal effect, while the Si substrate-side pumping at 780 nm leads to 21.3% THz emission suppression by photodoping. Moreover, the THz enhanced modulation efficiency peaks at an Ir layer thickness of 1.2 nm. Our work demonstrates an all-optical controllable Si-based THz source, providing critical insights for the design of next-generation on-chip THz functional devices.

## 1. Introduction

Terahertz (THz) radiation lies between microwaves and infrared, exhibiting characteristic spectral fingerprints of elementary excitations in solids, molecular rotations/vibrations, and biological macromolecules [1,2,3,4,5,6,7,8,9,10]. Featuring broad bandwidth, low photon energy, strong penetration, and unique spectroscopic identification ability, THz radiation shows great potential in material characterization, wireless communications, biomedical imaging, and security inspection [11,12,13,14]. Efficient generation of broadband coherent THz waves is essential for these applications. Conventional schemes such as photoconductive antennas and nonlinear electro-optic crystals are restricted by intrinsic phonon resonances of materials [15,16,17,18]. By contrast, gas plasma sources provide an ultrabroad bandwidth but rely on high-energy femtosecond lasers and are incompatible with on-chip integration [19,20,21]. Therefore, developing novel THz emitters with high efficiency, spectral tunability, and easy integrability has become an urgent demand in the THz field.

In recent years, the integration of THz technology and spintronics has established the rapidly developing field of THz spintronics [22,23]. THz radiation acts as an effective probe for the microscopic interactions between electron spins, magnons, phonons, and intra-band transitions, revealing fundamental physics such as giant magnetoresistance, tunnel magnetoresistance, and the spin Seebeck effect [22,24,25,26]. Notably, nanoscale heterostructure spintronic THz emitters (STEs) based on magnetic/metals with strong spin–orbit coupling have attracted extensive attention by utilizing ultrafast spin dynamics [27,28,29,30]. STEs feature a simple, etching-free structure, high stability, and low cost, while producing ultrabroadband THz pulses with tunable polarization and amplitude via external fields [31,32,33,34]. Moreover, STEs have a high optical damage threshold, allowing them to withstand high-power, high-repetition-rate laser excitation, which is essential for achieving strong-field THz emission and practical applications [35,36,37,38]. Benefiting from broadband metallic absorption, they can be pumped by femtosecond lasers over 400–1600 nm [39,40]. STEs have been combined with advanced techniques to study ultrafast dynamics, and their nanoscale thickness allows seamless on-chip integration [41,42], which is critical for next-generation THz optoelectronic chips.

The broadband THz emission properties of FM/NM heterostructures have been extensively studied. In 2013, Kampfrath et al. compared THz emission from Fe/Au and Fe/Ru bilayers [27]. In 2016, Seifert et al. optimized FM/NM bilayer and trilayer structures and examined the dependence of THz emission amplitude on total film thickness [28]. In 2017, Wu et al. demonstrated that THz emission from FM/NM bilayers is highly sensitive to individual layer thicknesses [43]. Regarding material selection, Co_20_Fe_60_B_20_ (CoFeB) as the FM layer yields stronger THz emission than Fe, Co, or CoFe [44,45]. The performance of STEs not only depends strongly on the type and thickness of FM and NM layers but also on the substrate [46,47]. Recently, combining STEs with semiconductors has attracted growing interest for further performance optimization [48,49]. Although most STEs are fabricated on MgO or quartz substrates, the dominant role of silicon in modern electronics makes the development of silicon-compatible STEs highly promising for practical applications [50]. Therefore, a systematic understanding of silicon-based STEs is essential for exploring high-performance THz functional devices.

In this work, we fabricate a CoFeB/Ir heterostructure on a silicon substrate, achieving pronounced coherent THz emission. For the first time, we realize efficient enhancement and modulation of its THz emission under continuous-wave (CW) optical pumping at room temperature, where the CW laser-induced nonequilibrium photothermal driving leads to nonlinear THz amplitude modulation. The THz emission enhancement arises from the competition between the photothermal effect and the photodoping effect, attributed to a change in conductivity. Additionally, the emitted THz pulse amplitude can be effectively modulated by tuning the Ir layer thickness. These results demonstrate that the Si-based CoFeB/Ir heterostructure is a promising platform for all-optical controllable THz emission, with great potential for next-generation on-chip THz functional devices.

## 2. Sample and Experimental Setup

A CoFeB/Ir heterostructure was fabricated on a Si substrate via direct current (DC) magnetron sputtering. The sputtering system was maintained at a base pressure below 4 × 10^−5^ Pa, with an argon (Ar) working pressure of 0.5 Pa throughout the deposition process. A 4 nm thick Ta buffer layer was first deposited on a thermally oxidized Si substrate to mitigate interfacial stress between the heterostructure and substrate, thereby improving thin-film quality. Subsequently, an 8 nm thick ferromagnetic CoFeB layer was deposited on the Ta buffer layer, followed by nonmagnetic Ir layers with varying thicknesses, d (Ir) = 0.4, 0.8, 1.2, 1.6, 2.4, 4, 6, 8, and 10 nm. The sputtering rates for Ta, CoFeB, and Ir were 0.27, 0.37, and 0.18 Å/s, respectively. The Si substrate used in this work is single-side polished, with a resistivity of 10 kΩ·cm, a thickness of 500 μm, and <100> crystallographic orientation.

**Figure 1:**
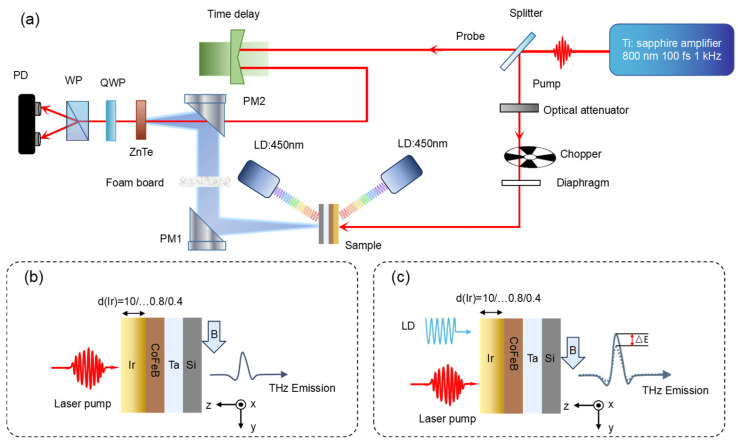
(**a**) Schematic diagram of the THz emission spectroscopy experimental setup. The red line denotes the propagation path of the pump and detection beams, while the blue region represents the THz radiation. (**b**) THz emission from Si-based CoFeB/Ir heterostructures without CW laser irradiation. An in-plane magnetic field H = ±125 mT is applied along the y-axis, and a fs laser induces THz emission polarized along the -x direction. (**c**) Anomalous enhancement of THz emission from the Si-based CoFeB/Ir heterostructure under CW laser irradiation.

Figure 1a shows a transmission-mode THz emission spectroscopy system. A Ti:sapphire femtosecond laser amplifier delivered laser pulses with a central wavelength of 800 nm, a repetition frequency of 1 kHz, and a pulse width of 120 fs. The femtosecond laser pulse was split by a beam splitter into a pump beam (for sample excitation and THz pulses generation) and a probe light (for THz pulses detection). The pump beam was mechanically chopped at 500 Hz and was normally incident on the CoFeB/Ir heterostructure, placed in a steady magnetic field (H = 125 mT, along the y-axis) perpendicular to the pump beam propagation, as shown in Figure 1b. THz pulses generated by the heterostructure were focused onto a 1 mm thick ZnTe electro-optic crystal via off-axis parabolic mirrors. The probe beam passed through an optical delay line and was collinearly incident with the focused THz beam on the ZnTe crystal. Driven by the THz pulse, the ZnTe crystal exhibited refractive index birefringence, leading to a change in the polarization state of the probe beam that followed the time-domain waveform of the incident THz pulse. A quarter-wave plate, a Wollaston prism, and a balanced-bridge photodetector were used to collect the THz time-domain signal as a function of the time delay between the probe light and THz pulses. All experiments were performed at room temperature in dry air to eliminate THz absorption by atmospheric water vapor. A CW laser diode (LD) was used to investigate the modulation of THz generation under laser illumination. As shown in Figure 1c, THz emission spectroscopy was integrated with a CW laser light (oblique incidence angle at 40°) from either the front or back side as an external stimulus. CW laser pumping directly excited the heterostructure on the substrate, with the excited state imprinted onto the emitted and transmitted THz signals. The spot size (1/e^2^ diameter) of the 800 nm femtosecond laser is approximately 2.5 mm, and that of the CW lasers (wavelengths: 450, 520, 639, 780 nm) is approximately 3 mm. Both were measured using the knife-edge method. The reflectance of the heterostructure was measured at about 45% for the 800 nm femtosecond laser and about 35.7% for the 450 nm CW laser.

## 3. Results and Discussion

**Figure 2:**
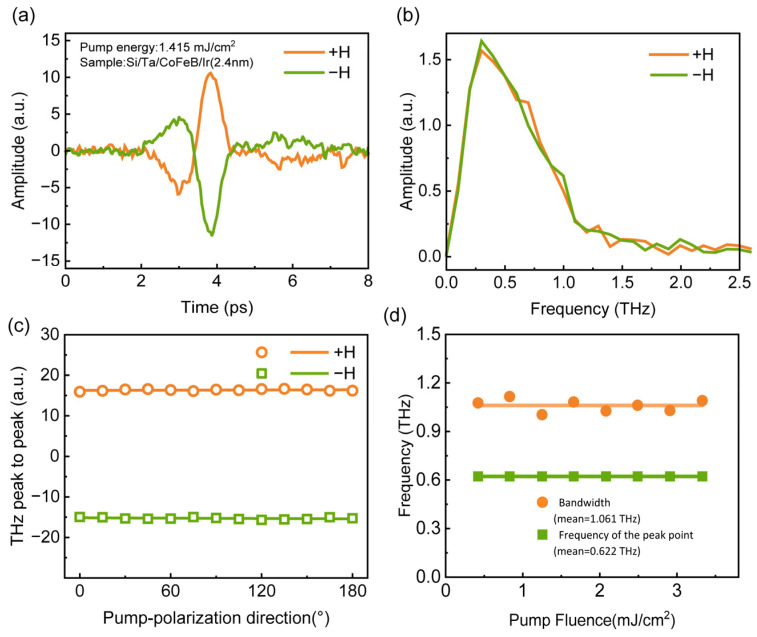
(**a**) THz time-domain pulses and (**b**) corresponding frequency spectra of Si-based CoFeB/Ir(2.4 nm) heterostructure under ±H = 125 mT with a pump laser fluence of 1.42 mJ/cm^2^. (**c**) THz peak-to-peak amplitude of CoFeB/Ir(2.4 nm) as a function of linear polarization angle of the pump laser. (**d**) Full width at half maximum (FWHM, mean value: 1.061 THz) and peak frequency (mean value: 0.622 THz) of THz spectra of CoFeB/Ir(2.4 nm) under different femtosecond laser pump fluences.

Figure 2a and Figure 2b show the time-domain waveforms of THz pulses generated by a Si-based CoFeB(8 nm)/Ir(2.4 nm) heterostructure under 800 nm femtosecond laser excitation and corresponding frequency spectra with an effective bandwidth covering 0–2.5 THz, respectively. The femtosecond laser pulses were incident on one side of the heterostructure. Reversing the direction of the applied magnetic field inverted the polarity of the THz pulse signal, with no changes in signal amplitude or spectral characteristics. This result confirms that the THz emission is directly correlated with the magnetic order of the heterostructure. Since the CoFeB/Ir is fabricated on the Si substrate, flipping the sample to investigate the symmetry relationship between THz radiation and the sample via substrate-side femtosecond laser excitation was not feasible.

In STEs, the FM layer is photoexcited by ultrashort visible-to-near-infrared laser pulses, driving electrons into a nonequilibrium high-energy distribution [51]. The excited electrons undergo spin-dependent relaxation and transport in the FM layer, generating a spin-polarized super-diffusive current [52,53]. When the spin current ($j_{s}$) is injected into the NM layer, it is converted into a charge current ($j_{c}$) via the inverse spin Hall effect (ISHE), the inverse Rashba–Edelstein effect (IREE), or a combination of them, with the conversion efficiency governed by the spin–orbit coupling strength of the NM layer. The induced jc has a picosecond-scale duration [27], which emits THz-frequency electromagnetic radiation. This spin-to-charge conversion (SCC) mechanism follows the relation $j_{c}\propto j_{s}\times M$ (M is the magnetization direction of the FM layer). As jc is perpendicular to M, the polarization direction of the emitted THz pulse is orthogonal to the applied magnetic field. The THz signal from the CoFeB layer via magnetic dipole radiation is typically one order of magnitude weaker than that generated by the SCC mechanism.

Figure 2c shows the THz emission of CoFeB/Ir(2.4 nm) as a function of pump laser polarization angle (0–180°, adjusted by a half-wave plate). The peak-to-peak amplitude of the THz time-domain signal remains nearly constant at all polarization angles, and this behavior is unchanged upon magnetic field reversal. Figure 2d depicts the THz spectral peak frequency and full width at half maximum (FWHM) with pump fluence varying from 0.42 to 3.3 mJ/cm^2^ (THz spectra recorded for each fluence; see Supporting Information Figure S1). The peak frequency stabilizes at ~0.622 THz and the average FWHM at ~1.061 THz, showing the THz spectrum is insensitive to pump fluence. The CoFeB/Ir heterostructure thus exhibits robust THz emission under varying pump fluences and polarizations, verifying the feasibility of integrating STEs with Si-based devices and their compatibility with THz time-domain spectroscopy requirements.

**Figure 3:**
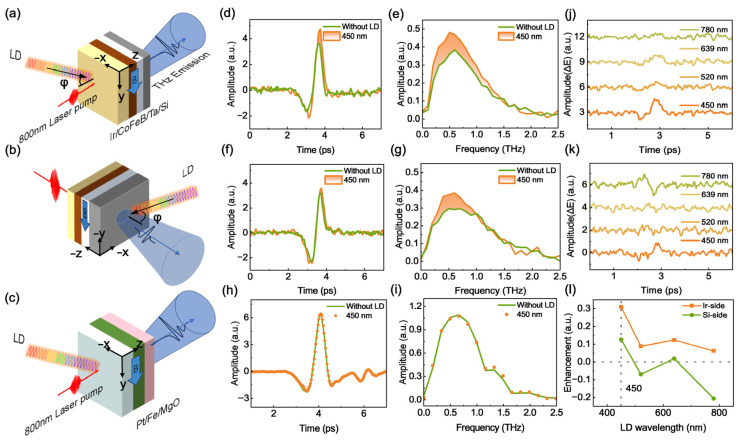
Schematic of the measurement geometry for Si-based CoFeB/Ir(2.4 nm) heterostructures illuminated by CW LDs of different wavelengths from the (**a**) front (Ir) and (**b**) back (Si) side. (**c**) Schematic of the front (Pt) side illumination of the MgO/Fe(3 nm)/Pt(3 nm) with a CW LD of different wavelengths. The red line represents the 800 nm femtosecond pump laser, while the colored curve corresponds to continuous-wave (CW) optical irradiation. (**d**) THz TDS and (**e**) corresponding frequency spectra of Si-based CoFeB/Ir(2.4 nm) under front-side illumination, with and without 450 nm CW LDs irradiation. (**f**) THz TDS and (**g**) corresponding frequency spectra under back-side illumination, with and without 450 nm CW LDs irradiation. (**h**) THz TDS and (**i**) corresponding frequency spectra of the MgO/Fe/Pt bilayer under front-side illumination, with and without 450 nm CW LDs irradiation. (**j**) THz signal change (ΔE) of Si-based CoFeB/Ir under front-side and (**k**) back-side illumination by CW LDs for different wavelengths. (**l**) THz signal modulation (ΔE/E_0_) of Si-based CoFeB/Ir as a function of the CW LDs wavelength.

During femtosecond laser-induced THz generation, a CW LD irradiated the CoFeB/Ir heterostructure at an oblique angle of 40°, from the front (heterostructure side) or back (substrate side), as shown in Figure 3a and Figure 3b, respectively.

Figure 3d shows the THz temporal waveforms of Si-based CoFeB/Ir(2.4 nm) without (green curves) and with 450 nm CW laser front-side pumping (orange curves), at a power density of 1.415 W/cm^2^. Under CW laser irradiation, the coherent generation of THz waves is significantly enhanced. As shown in Figure 3f, the heterostructure’s THz emission is also enhanced under back-side CW laser pumping. To verify if the enhancement is broadband or narrowband, the time-domain signals were converted to frequency-domain amplitude spectra (Figure 3e,g), which clearly confirm a broadband THz-emission amplification effect. A control experiment was conducted to investigate this anomalous enhancement effect (Figure 3c). Figure 3h and Figure 3i show the THz temporal waveforms and corresponding spectra of a MgO-based Fe/Pt heterostructure, respectively. No THz emission enhancement was observed for the MgO-based Fe/Pt heterostructure.

To further clarify the mechanism of THz emission enhancement, we pumped the Si-based CoFeB/Ir heterostructure with CW LDs at 450 nm, 520 nm, 639 nm, and 780 nm. To evidence the change in temporal modulation, Figure 3j shows the differential THz electric field ΔE(t), defined as the THz emission signal E_0_ (t) (without CW pumping) minus the signal E(t) under CW irradiation, at the above wavelengths. The enhancement of THz radiation decreases with the increase in CW laser wavelength for front-side CW pumping. Figure 3k shows the ΔE(t) for back-side CW pumping. In contrast to the front CW pumping, the THz emission is reduced at 780 nm. Figure 3l summarizes the ΔE/E_0_ as a function of CW pumping wavelength. For front-side pumping, ΔE/E_0_ peaks at 30.9% at 450 nm and decreases with increasing CW pump wavelength, with all positive ΔE/E_0_. For back-side pumping, ΔE/E_0_ is a positive 12.6% at 450 nm, while ΔE/E_0_ becomes negligible at wavelengths of around 520 nm and 639 nm, and becomes approximately −21.3% at 780 nm CW laser pumping.

This modulation behavior can be explained by the conductivity variation in the Si-based heterostructure. As previously reported, photothermal excitation induces enhanced THz transmission in Si-based samples [54], which is attributed to carrier scattering. CW laser irradiation first heats the metallic heterostructure, and then the generated heat transfers to the Si substrate, causing indirect thermal excitation of the substrate. With increasing LD power, the thermally induced increase in carrier density is limited, while the carrier scattering dominates and raises the Si resistance, thus enhancing THz transmission. Based on the Drude model [55], an elevated carrier scattering rate reduces the real part of the complex conductivity in the THz range, leading to enhanced THz emission [56,57]. In contrast, MgO has an ultra-wide bandgap (~7.8 eV) and negligible intrinsic absorption of 450 nm photons, resulting in almost no photothermal modulation. Our experimental results confirm that such photothermal enhancement of THz emission is a unique characteristic of Si-substrate-based STEs.

Notably, the photon energy of 450 nm (~2.76 eV) exceeds the Si bandgap (~1.1 eV) and can be absorbed by the surface layer of the Si substrate. Thus, the CW laser not only indirectly heats the sample but also induces a photodoping effect. Photo-injected carriers in the Si substrate enhance its electrical conductivity, which in turn reduces the transmission of THz radiation [55,58]. Typically, the integration of Si substrates with 2D materials in such heterostructures will further degrade THz transmission [59,60,61]. Our experimental results show that the CW laser’s modulation effect strongly depends on its wavelength and incident direction, which stems from the competition between photothermal and photodoping mechanisms under different conditions.

For front-side CW laser illumination, incident energy of all wavelengths is directly absorbed by the metallic layers of the heterostructure, generating a strong localized photothermal effect that reduces the Si substrate’s THz conductivity and dominates the observed THz emission enhancement. For back-side illumination, modulation originates from the competition between photothermal and photodoping effects in the Si substrate. At 450 nm, the photothermal effect outweighs the photodoping effect, leading to THz emission enhancement. As the wavelength increases—especially at ~780 nm near the Si bandgap—the photothermal effect is significantly weakened. Instead, photodoping becomes dominant, where a high density of photoexcited nonequilibrium carriers elevates the Si substrate’s THz conductivity, enhancing THz wave absorption and thus suppressing the THz transmission signal.

**Figure 4:**
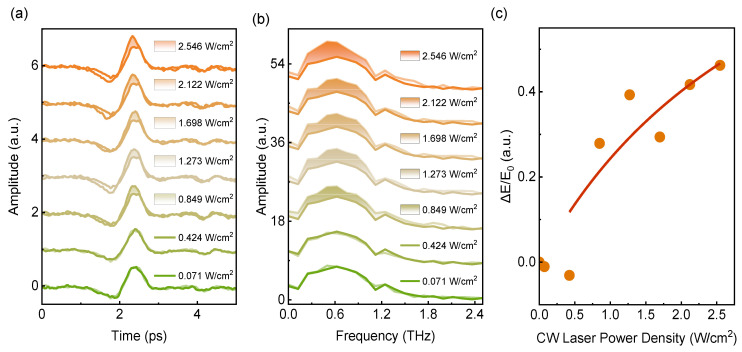
(**a**) THz time-domain signals emitted from Si-based CoFeB/Ir(2.4 nm) heterostructure without and with photothermal excitation under different power densities of 450 nm CW laser. (**b**) Corresponding frequency spectra of the time-domain signals in (**a**). (**c**) ΔE/E_0_ as a function of 450 nm CW laser power density. All time-domain signals were recorded at the same position on the sample. The CW laser power density was increased monotonically from low to high (0→0.071→0.424→0.849→1.273→1.698→2.122→2.546 W/cm^2^). After each power change, a waiting time of 2 min was allowed for thermal equilibrium before recording the signal. The red line serves as a guide, while the orange spheres represent the experimental data.

Next, we investigate the effect of CW LD pumping power on THz emission modulation. Figure 4a,b show the THz temporal waveforms and corresponding spectra from the Si-based CoFeB/Ir(2.4 nm) heterostructure under front-side 450 nm CW LD pumping at different power densities in comparison with the unpumped state. As shown in Figure 4c, ΔE/E_0_ of Si-based CoFeB/Ir(2.4 nm) heterostructure exhibits a nonmonotonic variation with increasing CW LD power density. ΔE/E_0_ decreases when the CW pump power density is lower than 0.424 W/cm^2^, while, above this critical value, ΔE/E_0_ rises rapidly and monotonously before gradual saturation. At the maximum power density of 2.546 W/cm^2^, ΔE/E_0_ reaches a peak value of 46%. This nonmonotonic behavior stems from the transition of the dominant carrier-scattering mechanism under photothermal excitation with varying optical power. At relatively low pumping power, a moderate increase in carrier concentration raises the electrical conductivity and suppresses THz emission, corresponding to the dip in ΔE/E_0_ at ~0.424 W/cm^2^ in Figure 4c. With further increases in CW LD power, the photothermal effects strongly modulate carrier transport through additional scattering processes. The applied photothermal field may perturb the lattice periodic potential and enhance the carrier scattering, which reduces the electrical conductivity and thus leads to the observed enhancement of THz emission. Our operating conditions are safely within the damage-free regime [35,46]; detailed parameters are summarized in Table 1 below.

**Figure 5:**
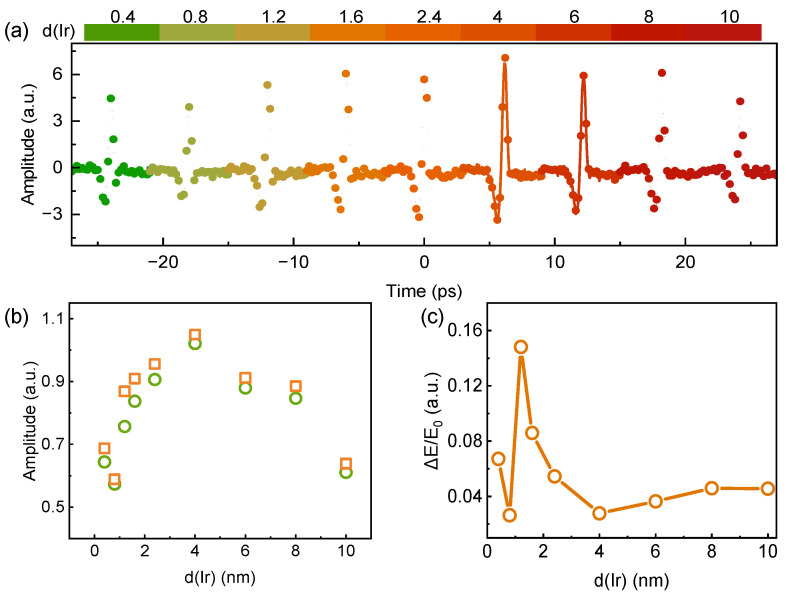
(**a**) THz time-domain signals from Si-based CoFeB/Ir(x) heterostructures with different Ir layer thicknesses, measured with (circular dots) and without (solid lines) 450 nm CW LD irradiation. The orange squares represent the THz peak values under 450 nm CW optical irradiation, while the green circles denote the THz peak values without 450 nm CW irradiation. (**b**) THz pulse peak amplitude as a function of Ir layer thickness. (**c**) ΔE/E_0_ as a function of Ir layer thickness d(Ir) under 450 nm CW LD pumping.

Finally, we investigated CW laser-modulated THz emission from Si-based CoFeB(8 nm)/Ir(x) heterostructures with Ir layer thicknesses x = 0.4–10 nm. Figure 5a shows the THz time-domain emission signals of each sample, measured under a fixed H = 125 mT and femtosecond pump fluence of 1.415 mJ/cm^2^, with and without 450 nm CW laser excitation. The circles represent THz emission signals under 450 nm CW laser irradiation; all of them have a higher intensity than the THz emission without CW laser irradiation (lines). Figure 5b shows the THz pulse peak amplitude as a function of Ir layer thickness. According to the thickness dependence of THz emission, the propagation length of the spin current can be estimated [62]. Figure 5c shows ΔE/E_0_ as a function of Ir layer thickness. ΔE/E_0_ reached a maximum of 0.15 when Ir thickness was 1.2 nm, indicating the most pronounced CW laser-enhanced modulation of THz emission at this thickness. This result shows that CW-laser-enhanced THz emission from Si-based heterostructures depends not only on laser wavelength and power but also critically on the FM/NM heterostructure’s thickness.

## 4. Conclusions

In summary, we demonstrate efficient photothermal modulation of THz emission from a Si-based CoFeB/Ir spintronic heterostructure. The THz emission originates from the inverse spin Hall effect, and its enhancement under CW laser illumination originates from a Si-specific photothermal mechanism. At 450 nm, the signal enhancement saturates with increasing optical power, achieving a maximum enhanced modulation of ~46%. By varying the Ir layer thickness, the strongest modulation is achieved at 1.2 nm. This work provides a high-performance, optically controllable Si-based THz source, offering important implications for integrated and tunable THz functional devices.

## Figures and Tables

**Table 1:** Summary of the pulsed and CW laser excitation parameters used in this work.

Laser Type	Beam Area (cm^2^)	Min. Fluence/Power Density	Max. Fluence/Power Density	Min. Average Power (mW)	Max. Average Power (mW)
800 nm (pulsed)	0.0491	0.42 mJ/cm^2^	3.30 mJ/cm^2^	10.3	81.0
450 nm CW	0.0707	0.071 W/cm^2^	2.546 W/cm^2^	5	180

## Data Availability

The original contributions presented in this study are included in the article. Further inquiries can be directed to the corresponding authors.

## Supplementary Materials

The following supporting information can be downloaded at: https://www.mdpi.com/article/10.3390/nano16090530/s1, Figure S1: Terahertz (THz) frequency-domain signals of the sample under different pump fluences.
