# Supplementary material for: Enhanced Modulation of Terahertz Generation in Optically Pumped Silicon-Based CoFeB/Ir Heterostructures
Source: Nanomaterials (Basel). 2026 Apr 28;16(9):530. doi: 10.3390/nano16090530 (PMC13164665; doi:10.3390/nano16090530)
Supplement: Supplementary file 1 [file nanomaterials-16-00530-s001.zip › nanomaterials-4282134-supplementary.pdf]

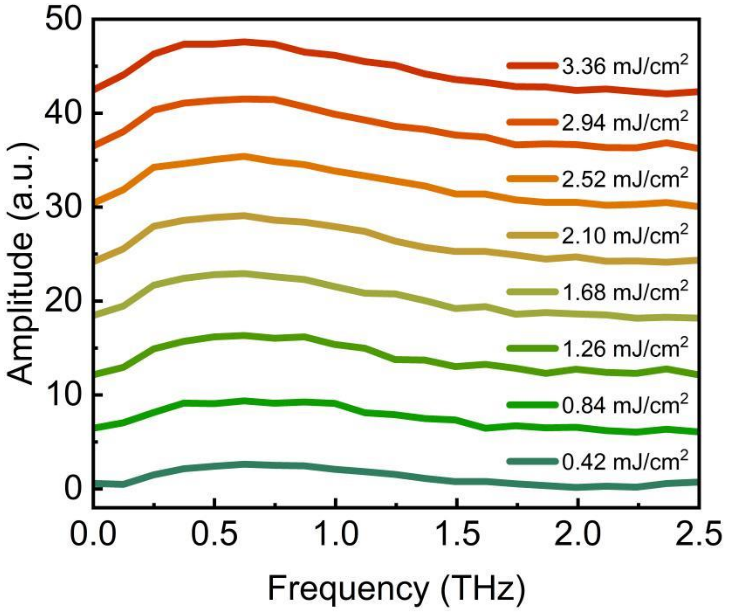

Figure S1. Terahertz (THz) frequency-domain signals of the sample under different pump fluences. The curves correspond to pump fluences from 0.42 to 3.36 mJ/cm² with a color gradient from green to red. The THz amplitude increases monotonically with pump fluence, and all spectra exhibit a distinct peak at around 0.622 THz for all pump fluences.
